# Multidisciplinary approach to the study of large-format oil paintings

**DOI:** 10.1038/s41598-023-28777-9

**Published:** 2023-02-07

**Authors:** P. Calderón-Mesén, D. Jaikel-Víquez, M. D. Barrantes-Madrigal, J. Sánchez-Solís, J. P. Mena-Vega, J. Arguedas-Molina, K. Ureña-Alvarado, G. Maynard-Hernández, L. Santamaría-Montero, M. Cob-Delgado, E. Angulo-Pardo, Felipe Vallejo, M. I. Sandoval, A. M. Durán-Quesada, M. Redondo-Solano, O. A. Herrera-Sancho

**Affiliations:** 1 Centro de Investigación en Estructuras Microscópicas, Universidad de Costa Rica, 2060 San Pedro, San José Costa Rica; 2 Instituto de Investigaciones en Arte, Universidad de Costa Rica, 2060 San Pedro, San José Costa Rica; 3 Facultad de Microbiología, Universidad de Costa Rica, 2060 San Pedro, San José Costa Rica; 4 Centro de Investigación en Enfermedades Tropicales (CIET), Universidad de Costa Rica, 2060 San Pedro, San José Costa Rica; 5 Escuela de Química, Universidad de Costa Rica, 2060 San Pedro, San José Costa Rica; 6 Escuela de Ingeniería Eléctrica, Universidad de Costa Rica, 2060 San Pedro, San José Costa Rica; 7 Escuela de Física, Universidad de Costa Rica, 2060 San Pedro, San José Costa Rica; 8 Diseño Gráfico, Sede de Occidente, Universidad de Costa Rica, 2060 San Ramón, Alajuela Costa Rica; 9 Department of History of Art, Cornell University, Ithaca, NY 14853 USA; 10 Escuela de Artes Plásticas, Universidad de Costa Rica, 2060 San Pedro, San José Costa Rica; 11 Instituto Costarricense de Investigación y Enseñanza, en Nutrición y Salud, 42250 Cartago, Costa Rica; 12 Grupo de Investigaciones en Estratigrafía, y Vulcanología (GIEV-Cumanday) y Departamento de Ciencias Geológicas de la Universidad de Caldas, Instituto de Investigaciones en Estratigrafía (IIES), Calle 65 # 26-10, 1700004 Manizales, Colombia; 13 Departamento de Geología, Facultad de Ciencias, Universidad de Salamanca, España, Plaza de los Caídos, s/n, 37008 Salamanca, Spain; 14 Escuela Centroamericana de Geología, Universidad de Costa Rica, 2060 San Pedro, San José Costa Rica; 15 Departamento de Física Atmosférica, Oceánica y Planetaria & Laboratorio para la Observación del Sistema Climático, Escuela de Física, Universidad de Costa Rica, 2060 San Pedro, San José Costa Rica; 16 Centro de Investigación en Contaminación Ambiental, Universidad de Costa Rica, 2060 San Pedro, San José Costa Rica; 17 Laboratorio de Investigación y Entrenamiento en Microbiología de Alimentos y Aguas (LIMA), Universidad de Costa Rica, 2060 San Pedro, San José Costa Rica; 18 Centro de Investigación en Ciencias Atómicas Nucleares y Moleculares, Universidad de Costa Rica, 2060 San Pedro, San José Costa Rica

**Keywords:** Environmental monitoring, Characterization and analytical techniques, Scanning electron microscopy, Environmental microbiology

## Abstract

Cultural heritage has become a keystone for comprehending our society, as it represents and reflects our origins, passions, beliefs and traditions. Furthermore, it provides fundamental information about specific temporary spaces, materials’ availability, technology, artist’s intention, and site weather conditions. Our aim was to develop a multidisciplinary approach with a main focus on investigating two Italian large-format paintings located in highly diverse environments such as the National Theater of Costa Rica. We monitored environmental conditions and quantified fungal aerial spores. Then, we determined regions of possible biodeterioration with the software *MicroorganismPattern* and used the software *PigmentArrangement* to elucidate the apparent colour of the paintings based on distribution and arrangement of the pigment crystals. Finally, we characterized eight genera of calcareous nannofossils found in the ground layers of the artwork. The former Men’s Canteen at the National Theater of Costa Rica presented a mean air temperature of 23.5 $^{\circ }$C, a relative humidity of 72.7% and a concentration of CO$_{2}$ of 570 ppm. The fungal aerial concentration was 1776 spores/m$^{3}$. The software *MicroorganismPattern* identified 32 sampling regions, out of which 11 were positive for microbial contamination. The software *PigmentArrangement* determined that the blue crystals (ultramarine pigment) had the shortest distances between themselves (29 $\upmu$m). Finally, the nanofossils identified enabled us to restrict the age of the material to a biostratigraphic interval ranging from Coniacian to Maastricthian ages. By using a multidisciplinary approach we were able to explore the diptych, suggest a set of minimally invasive perspectives in tropical environments to be used worldwide and obtain key information about the artist’s artistic process, materials used along with better understand its state of conservation.

## Introduction

Since ancient times, humanity has produced works of art and faced various challenges related to the production, conservation, and restoration of what we now refer to as cultural heritage. The ultimate quest to reconstruct the object, its history, the artist’s intention, its structural integrity and color changes, and the impact caused by climatological conditions lies not only in having a realistic understanding of the dynamics of the object’s materials but also of its surroundings and storage conditions. For this reason, the history of pictorial materials and their artistic techniques involves experimentation and creativity in both the artistic and scientific fields, as these areas and their characteristics should be considered while exploring the object in question^1^. It is essential to establish multidisciplinary approaches to the study of artworks all over the world since these cultural heritages are located in latitudes with very contrasting environmental or storage conditions as well as very divergent socioeconomic situations (low economic resources and lack of political interest); all of which will influence the state of conservation of the artefact. Therefore, the knowledge obtained from these studies can contribute to the establishment of worldwide public policies on cultural heritage and to ensure that future generations will have the opportunity to appreciate them.

**Figure 1:**
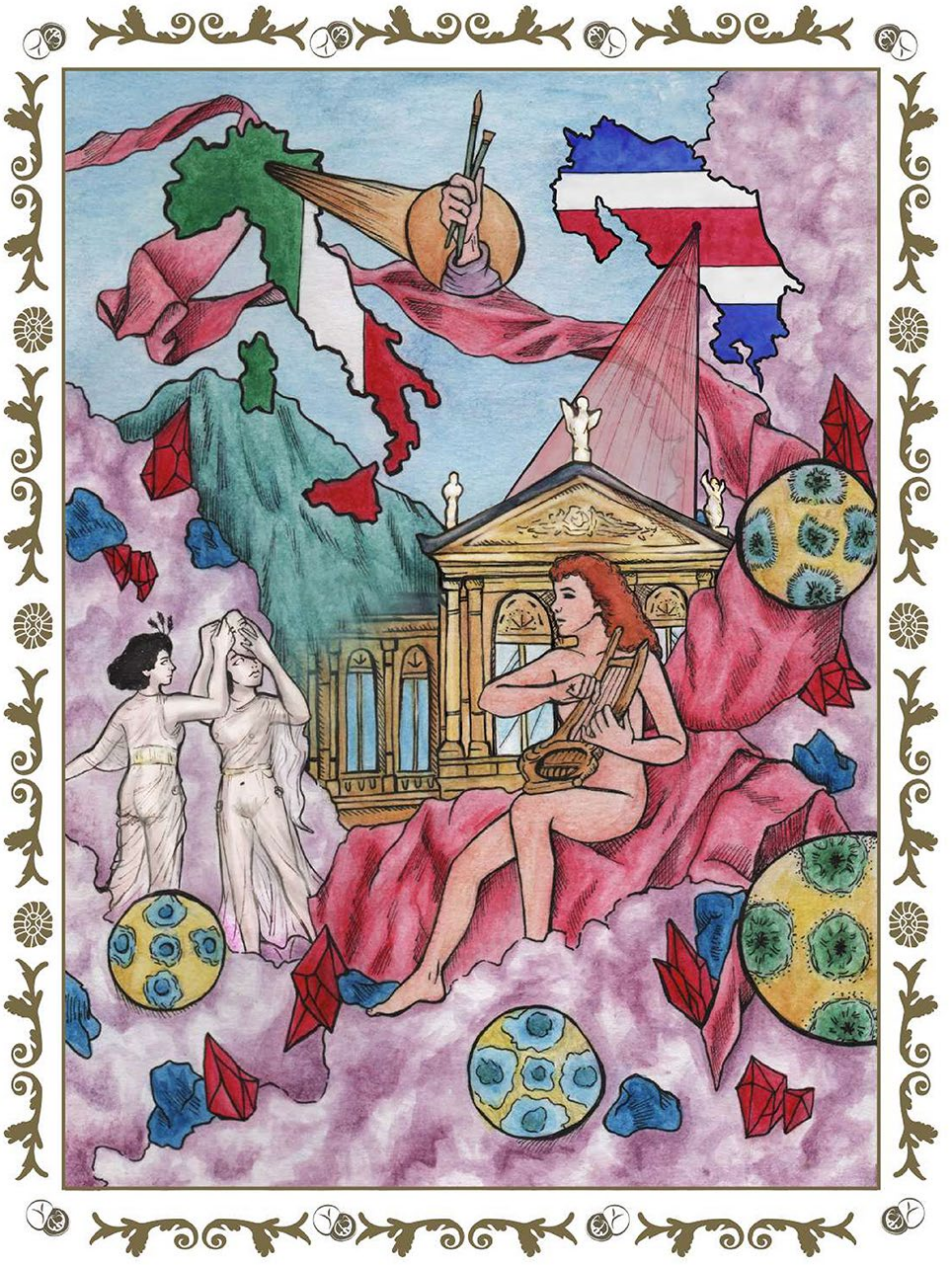
Uncovering the glittering past to cherish and protect the present of cultural heritage. A deeper study of the colour palette’s pigments arrangement, environmental conditions, microorganisms as well as nannofossils present in the first ground of the paintings was conducted along with different software. This research describes the historical context, origins, and tropicality of a large-format Italian diptych, *Musas I* and *Musas II*, located in Costa Rica’s National Theater. The figure was created by the authors following what is described in the Supplementary Information and using the following computer tools: Adobe Illustrator, version number 27.1 (https://www.adobe.com/la/products/illustrator.html) and Adobe Photoshop, version number 23.3.2 (https://www.adobe.com/la/products/photoshop.html).

The present study analyzes two large-format paintings, known as *Musas I* and *Musas II*, which decorate the ceiling of the chamber originally used as the Men’s Canteen room at the National Theatre of Costa Rica (NTCR) (built in 1891–1897). They embody the Greek god Apollo and the Muses at Mount Parnassus (see Fig. 1 for our artistic visualization). The two canvases were painted in Milan in 1897 by Carlo Ferrario (1833–1907), an Italian painter, theater decorator, and teacher who worked at the Academia di Brera, as well as the Teatro alla Scala. In a previous work, we determined the artist’s main color palette (used in these paintings) via the usage of software-based imaging techniques^2^. However, the goals of reaching deeper into the development of minimally invasive approaches that are accessible, efficient, and multidisciplinary to comprehend cultural heritages that are constantly changing alongside with its environmental conditions have remained elusive. Thus, to conduct a nonintrusive approach in these artworks, a technical examination of the painting and its environment is essential. Normally, paintings consist of many superimposed layers where the pigments and the binder interact in a varied and complex way causing color changes and discoloration; these are inherent to the artist’s selection of materials and the restoration interventions. Some of these changes can be attributed to microorganisms and weather conditions, as they can accelerate these processes of deterioration^3–5^, especially in tropical environments such as those found in Costa Rica. It is worth noting that the study of art conservation in tropical areas has been scarcely explored.

Henceforward, in our efforts to establish a multidisciplinary approach to the study of artworks we investigated the artistic palette used by Ferrario through four decisive and distinct approaches: probable and available trade routes, temporary availability of mines along with pigments, spectroscopic information of the chemical composition of samples extracted from his work, and direct comparison with the pigments recommended by Ferrario in his treaty published in 1931^6^. Additionally, by employing microscopic imaging techniques, in combination with a state-of-the-art software (*PigmentArrangement*), we elucidated the apparent colour of the artworks based on distribution and arrangement of the crystals in each pigment (chrome yellow, lead read, ultramarine, vermilion and viridian).

As previously stated, the materials in oil paintings not only interact with each other but also with their surroundings over time, which is why the monitoring of the artworks’ environment is imperative to better understand its state of conservation. Thus, we determined the fungal aerial spore concentration, and, with the usage of novel meteorological stations, we monitored temperature, humidity, light intensity, and CO$_{2}$ content in the chamber where the paintings are located. Then, we applied the software *MicroorganismPattern*, which uses ultraviolet technical photography, to distinguish possible areas of microbial growth in the paintings. The reliability of its measurements has an accuracy of approximately 11%, corresponding to a quantitative determination. This assessment provides an exciting opportunity to advance in our interpretation of an early detection of microbial contamination, while using a software as well as a systematic and minimally invasive approach. This is relevant when very few recent studies focus on image recognition of biodeterioration patterns particularly in paintings. For instance, recent research using machine learning to predict areas of deterioration based on information from cross-sections of samples (not based on the large-format images of the artworks) was used to study a tempera painting, *Celje Ceiling*, located in Old Counts’s Mansion, Slovenia^7^.

Lastly, we classified eight genera of calcareous nannofossils found in the ground layers of the paintings. *Musas I* and *Musas II* have four well defined layers: top paint layer, intermediate paint layer, second ground layer, and first ground layer^2^. The ground layers, also known as *gesso*, are preparatory and priming steps for the later oil-bound layers^8^; they are a combination of calcium carbonate materials, glue, and white pigments. In our previous research, we found that the white pigments used by the artist in this diptych were zinc white and lead white^2^. The calcium carbonate materials, known as chalk, consist of deep marine deposits, and they are characterized by the presence of calcareous nannofossils^8,9^. The study of the biodiversity and abundance of these nannofossils could provide information about the geographical distribution of these organisms^10,11^, and therefore, it can provide clues to identify the origin of the materials along with determining a chalk trade market source^12^. Just as studies of nannofossils have provided indications of the origin of objects of cultural heritage, such as medieval mortars^13^ and Egyptian hieroglyphic^14^. Ground materials and white pigments could have different optical properties^9,15^, and the fabrication process of lead white could result in subtypes of the crystalline lead carbonate in the pigment. Some of the forms might be cerrusite and hydrocerussite that can change the opacity and brightness of the painting^15^. The ground layers in combination with the colored pigments layers and the canvas materials create a complex stratigraphic structure that composes the artwork. In general, these findings regarding ground layers enhance our understanding of the nature of the paintings.

The study’s objective was to examine an oil painting on canvas diptych from a unique interdisciplinary perspective, as it implements history, art, and applied sciences to cultural heritage. Our research focuses on analyzing the composition of substances that the artist employed to create the paintings and the journey of the artwork—considering that it was created in Italy and later transported to Costa Rica—to provide tools for the study of priority areas of intervention to conserve this cultural heritage. The following section describes the results and their respective discussion of the proposed methodology for the study of artworks focusing on environmental conditions, colour palette, microbial colonization and nannofossils in Ferrario’s diptych. Finally, the conclusions of our study along with the recommendations are presented. In the “Methods” section, all the characteristics are established to achieve the evidence of the reported results.

## Results and discussion

### In search of the origins of the artworks

**Figure 2:**
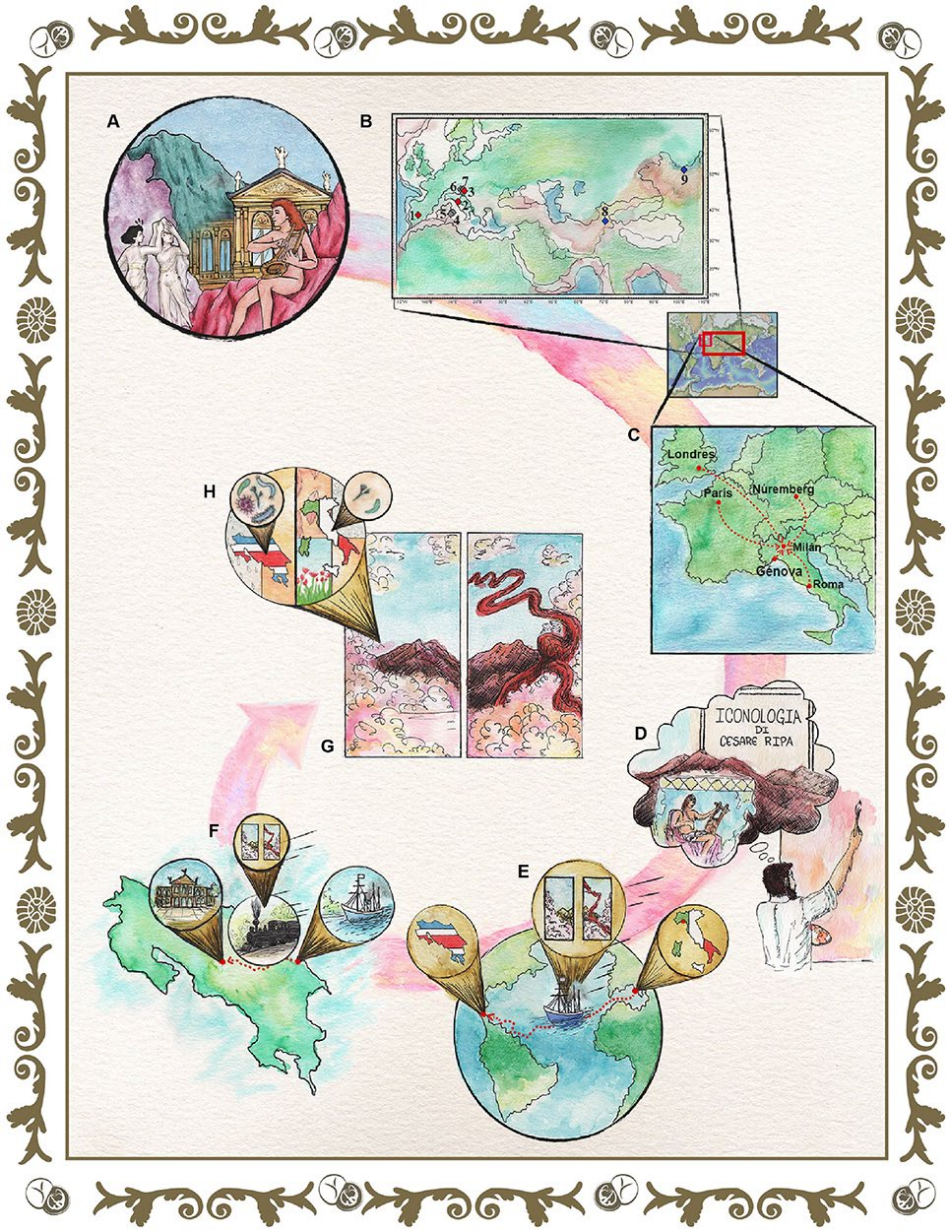
From the origin to the present of paintings. To understand the context of (**A**) the *Musas I* and *Musas II* oil paintings, this study includes information of (**B**) the probable mines from which the minerals were extracted for the pigments’ elaboration used in the paintings, (**C**) the trade routes that took them to Milan, where the artist Carlo Ferrario created the artworks, inspired by classical iconographic representations such as those established by Cesare Ripa (**D**). Subsequently, we discover that the works were transported to Costa Rica by sea (**E**) from the port of Genoa to Puerto Limón, (**F**) where they were subsequently transferred by rail to the National Theater of Costa Rica, in the country’s capital, San José. There, those paintings (**G**) still decorate the Theater and are exposed to weather conditions that differ from those in Europe. This difference in the environment surrounding the paintings has led to their present deterioration, also produced by the presence of contrasting microorganisms (**H**). The figure was created by the authors following what is described in the Supplementary Information and using the following computer tools: Adobe Illustrator, version number 27.1 (https://www.adobe.com/la/products/illustrator.html) and Adobe Photoshop, version number 23.3.2 (https://www.adobe.com/la/products/photoshop.html).

Carlo Ferrario, a well-known theater decorator, painted the two canvases with pigments that were available in the Milan art market during the late nineteenth century, as they came from various European mines and paint manufacturers^1,16^ (Fig. 2). We have verified that this diptych contains lead red, viridian, ultramarine, vermilion, chrome yellow, zinc white, and lead white^2^. Most of these pigments are recommended by Ferrario in his treatise *La tecnica della pittura ad olio ed a pastello*, in which he reveals the working methodology and formulas that he prefers in order to produce a wide variety of colours in oil painting^6^. According to a bibliographic and geographical review, the source material for the main pigments came from different possible mines around the world (Fig. 2B, the location of the mines is indicated in figure caption). The lead and zinc used in the lead white and zinc white as well as other pigments have a close relation with galena minerals and their derivatives^17^. Moreover, they could come from other mines in Italy such as Distrito Minero SW Sardinia^18^, Distrito Minero SE in Sardinia^19^, Salafossa^20^, and Rabil Cueva del Predil^21^. Sources for red pigments mainly came from mercury, as they are associated with the Cinabrio mineral. This mineral probably was extracted from two mines in Italy: Mt. Amiata and Abbadia San Salvatore^22,23^. Nonetheless, other mines could have been involved in its extraction such as Almadén, Spain^24^ and Idrija, Slovenia^25^. In the case of the blue pigments, they were obtained from the Lazurite mineral. Moreover, the most probable mines for this mineral are Sar e Sang, Afghanistan^26^; Baikel Lake Slyudyanskii, Russia^27^; and Flor de los Andes, Chile^28^. Finally, to clarify, Ferrario used synthetic ultramarine pigments instead of natural ones^2^.

*Musas I* and *Musas II* were commissioned by the Government of Costa Rica in 1896 and, they were painted by Ferrario in Milan in 1897. The iconography of Ferrario’s diptych materializes the legacy of Cesare Ripa’s Iconologia (1593) and the influence of Neoclassicism in the representation of Apollo and the Muses at Mount Parnassus. Historically, this iconographic theme has been considered suitable for an opera house due to the association of the muses with artistic inspiration. Moreover, in ancient Greek culture, Apollo is the god of the arts; his aesthetic influence is evident in the NTCR (Fig. 2D). Ferrario’s paintings were shipped from Italy to Puerto Limón, Costa Rica. Then, a few months before the NTCR inauguration in 1897, they were transported to San José by railway (Fig. 2E,F). However, Ferrario’s paintings brought to Costa Rica not only classical iconographies and European pigments but also nannofossils older than Mount Parnassus itself (see below).

### Current location and state of conservation

#### Microbiological detection with computer program

**Figure 3:**
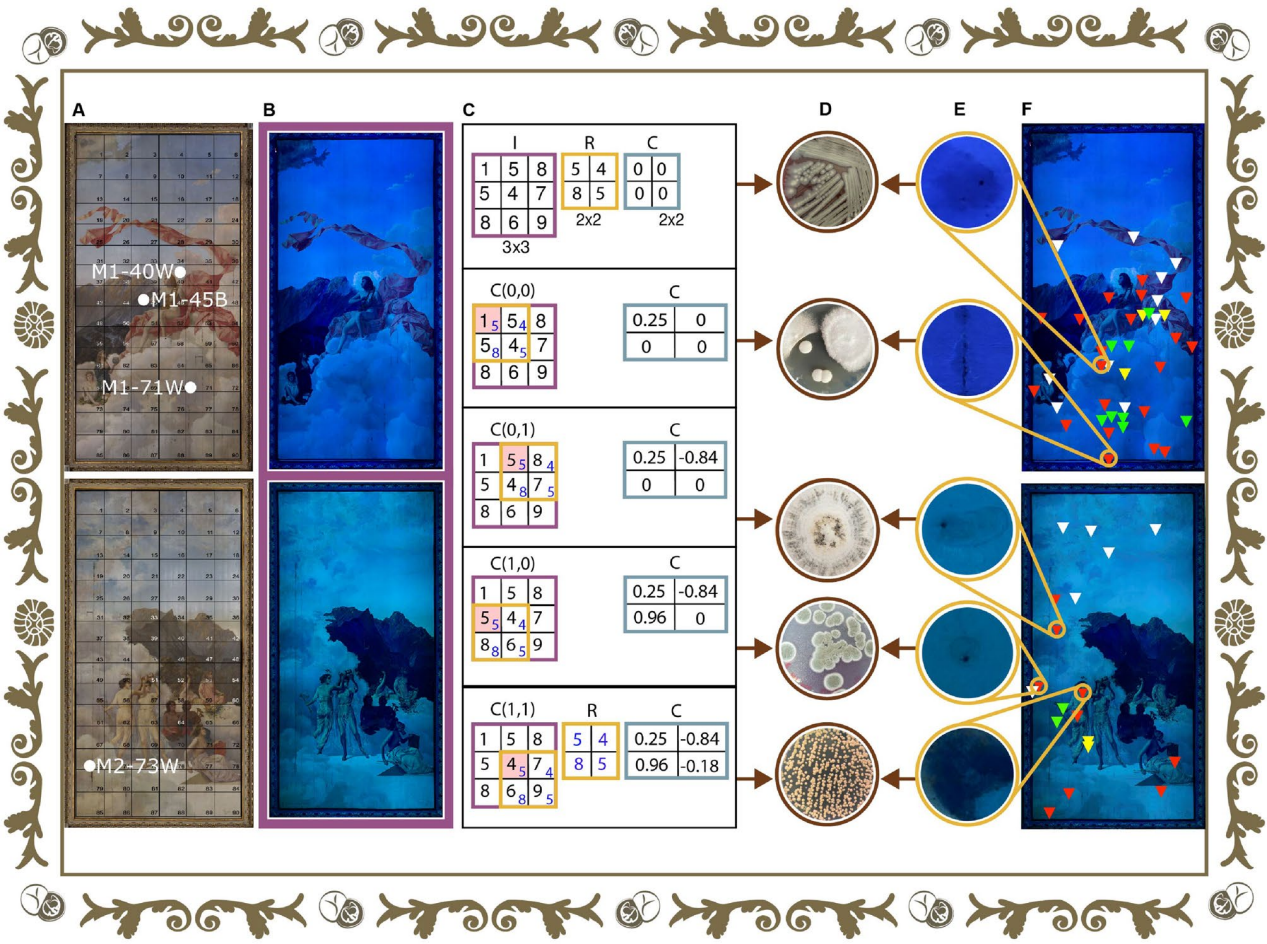
Determination of the biodeterioration areas of interest for direct microbiological sampling (**A**) Photographs of the paintings *Musas I* (top panel) and *Musas II* (bottom panel) in visual light with information of the samples of painting layer extracted for microscopical analysis. (**B**) Photographs of the paintings *Musas I* and *Musas II* in ultraviolet fluorescence (UVF) light. (**C**) First, the UVF photograph shown in (**B**) is discretized by its pixels and the information placed in the matrix called *I* (see purple colour). As described in the text, via meticulous observation, possible deterioration patterns are selected and placed on the artwork (see **E**). These patterns are used as a basis in the template matching method to carry out the object detection, and the pixels of the *R* matrix are accordingly assigned (see yellow colour). Finally, the *MicroorganismPattern*^29^ computational tool estimates matrix *C* using Eq. (1), as explained in “Methods” section. From the example indicated here, the value of the matrix *C* that is close to the value + 1 is the input *C(1,0)*. (**D**) Microorganisms isolated from the deterioration areas (from top to bottom: *Bacillus cereus*, fungi from family Polyporaceae, *Aspergillus* section *Nidulantes*, *Penicillium steckii*, *Cryptococcus uniguttulatus*) **(E)** Examples of significant types of deterioration observed in the painting (from top to bottom: types of deterioration); the areas were determined with UVF images. (**F**) Location of biodeterioration areas according to software (white) and *Musas I* visual biodeterioration areas: rounded dark blue (red), rounded light coloured (green), and rounded fluorescent (yellow); as for *Musas II*, visual biodeterioration areas: rounded faint coloured (red), rounded light coloured (green), and rounded dark blue (yellow). The figure was created by the authors following what is described in the Supplementary Information and using the following computer tools: Adobe Illustrator, version number 27.1 (https://www.adobe.com/la/products/illustrator.html) and Adobe Photoshop, version number 23.3.2 (https://www.adobe.com/la/products/photoshop.html).

The large-format diptych (total area of roughly 36.5 m$^{2}$) is an object that is alive and, at the same time, hard-to-reach for art professionals and restoration scientists. The large size and location (on the ceiling of the Men’s Canteen) of the artworks complicate the search for microorganisms. The most successful restoration and conservation approach is the one that follows a non-invasive technique such as systematically selecting regions of interest for microbiological sampling by means of software tools. For this reason, with the help of a novel software (*MicroorganismPattern*^29^) along with UVF images, we identified places with possible bacteria and fungi colonization. The software uses an algorithm that is based on a template matching method^30^. First, we analyze UVF photographs (Fig. 3B) in order to find a characteristic perceivably pattern and locate it. This pattern that resembles a probable type of deterioration, is established through cautious observation of the UVF photography of the artwork. The UVF image and the microorganism pattern are exemplified as matrix *I* and matrix *R* in panel C, respectively.

Next, a matrix *C* (Fig. 3C) is computed by the method of correlation coefficient between the pattern (*R*) and the entire artwork (*I*) (see “Methods” section for details). As a pilot study, we established the regions of interest through the accessible multidisciplinary software *MicroorganismPattern*. Then, we carried out two microbiological samplings in the large-format diptych. The most significant finding of this systematic sampling was that the software had a determination of approximately 11%; however, it is important to bear in mind that all the localities discovered were not sampled. From thirty-one areas of around 1 cm$^{2}$ that were swabbed, eighteen microorganisms were isolated and identified (see below).

#### Microbiological findings

In general, the paintings from the NTCR are exposed to constant interaction with human and environmental factors like relative humidity, temperature, and light that induce the deterioration processes^3^. Therefore, it is expected that a significant number of microorganisms may colonize and, eventually, deteriorate the general structure of each artwork^31^. The study of the role of some microbial populations that are present in these paintings is imperative for further elaboration of proper conservation strategies.

In both *Musas I* and *Musas II*, specific physical patterns that may correlate with deterioration were identified. Figure 3E presents five examples of these types of deterioration and the specific microorganisms isolated from each of them. A total of nineteen samples—thirteen during the first sampling date (including the negative control) and six in the second—were collected from *Musas I*. From the first sampling date, the bacterium *Bacillus cereus* was isolated from sampling area 63C3 and two bacteria (*Alicyclobacillus acidoterrestris* and *Kocuria rizhophila*) and a filamentous fungus (*Penicillium* section *Chrysogena*) from area 87D3 (deterioration area next to the painting’s frame). Both sampling areas presented black spots over brown stains. On the other hand, from the second sampling date, one bacterium and eight fungi were recovered from four sampling areas. The bacterium *Sphingomonas paucimobilis* was isolated from sampling area 71A3, *Cladosporium halotolerans*, *Cladosporium dominucacum* and *Rhodotorula glutinis* from area 77A1, two basidiomycetes from family Polyporaceae and a hyaline ascomycete from 87C3 and finally *C. halotolerans* and *Penicillium crustosum* from 88C1. The sampling areas 71A3 and 87C3 presented black spots over brown stains and the other two were areas with craquelures.

In the case of *Musas II*, thirteen samples (twelve during the first sampling date and one from the second) were gathered. From the first sampling date four microorganisms (one bacterium and three fungi) were isolated. *Aspergillus* section *Nidulantes* was isolated from sampling area 37D1, *Penicillium steckii* from 49A3/B3, *Cryptococcus uniguttulatus* from 56D1 and *B. cereus* from 61D1. Finally, *Staphylococcus lugdunensis* was isolated on the second sampling date from area 61C1. The areas 37D1 and 49A3/B3 presented black spots over brown stains and the other three areas discoloration. It is worth noting that no microbial contamination was detected on the negative control site and that different sampling areas were selected for each period.

**Figure 4:**
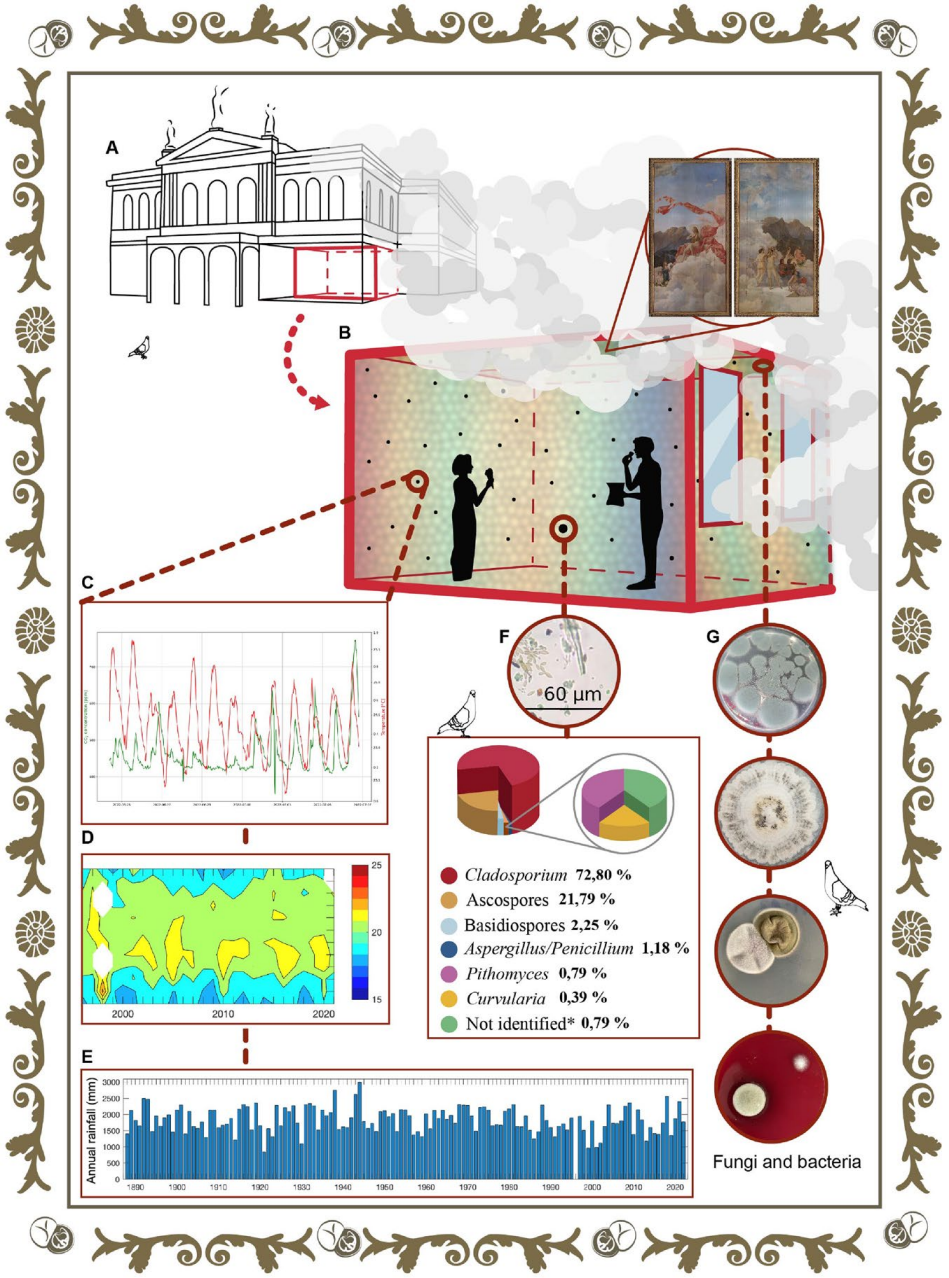
The tropical environmental conditions of the pictorial diptych living day by day (**A**) The NTCR building is located in the capital of the country, in a very busy place characterized by a constant flow of vehicles and people. (**B**) *Las Musas* reside in the ceiling of the former Men’s Canteen where they are exposed to environmental conditions and human activities. (**C**) Graphic that shows the monitoring of the environmental conditions (temperature, humidity, and CO$_{2}$) for a period of three months. The temperature variation since the year 2000 is shown in (**D**) and the amount of precipitation per year is observed in (**E**). A fungal aerial volumetric study was conducted in this room, where *Cladosporium* was the main fungal spore present (**F**). Finally, (**G**) shows some previously isolated and characterized bacteria and fungi from the paintings (from top to bottom: *Penicillium* section *Chrysogena*, *Aspergillus* section *Nidulantes*, *Cladosporium halotolerans* with *Cladosporium dominicanum* and *Cladosporium halotolerans* with *Penicillium crustosum*). The regions of microbiological interest were previously selected by means of the computational tool *MicroorganismPattern* and UVF photography. The figure was created by the authors following what is described in the Supplementary Information and using the following computer tools: Adobe Illustrator, version number 27.1 (https://www.adobe.com/la/products/illustrator.html) and Adobe Photoshop, version number 23.3.2 (https://www.adobe.com/la/products/photoshop.html).

*Penicillium* was the main fungal genus present in the paintings, as it was found in both sampling periods and both artworks, followed in importance by *Cladosporium* (found just in one sampling date). Both genera are widely described as contaminants of closed indoor environments^31^. The results obtained are coherent with the aerial volumetric examination at the Men’s Canteen (location of the paintings, see Fig. 4A,B,F,G). In this room, the main circulating fungal spores were conidia from the genus *Cladosporium* (1293 spores/m$^{3}$), *Ascospores* (387 spores/m$^{3}$), Basidiospores (40 spores/m$^{3}$), and conidia of either *Aspergillus* or *Penicillium* (21 spores/m$^{3}$) (cf. see Supplementary Information). Furthermore, it is worth noting that these fungi have enzymes (cellulases)^32,33^ that might be able to hydrolyze the components on the canvas and the wood from the paintings’ frame. Interestingly, one yeast was present in *Musas II* (*C. uniguttulatus*). This fungus is a normal inhabitant of pigeon’s (*Columba livia*) droppings^34^. Hence, its presence may be related to the park areas that are close to the NTCR. The blastospores may have been transported by wind to the paintings.

On the other hand, the isolation of bacteria confirms that the process of microbial decay in the artwork may be a complex one, as it may involve the interaction of different microbial communities; the findings from the 87D3 spot supports this idea (this sample area is the one on the botton border of *Musas I*, see Fig. 3F). All the bacterial genus that were identified (*Bacillus* spp., *Alicyclobacillus* spp., *Kocuria* spp., *Staphylococcus* spp., and *Sphingomonas* spp.) are microorganisms commonly present in the environment, as they have capacity to form endospores (*Bacillus* spp. and *Alicyclobacillus* spp.)^35^ or biofilms (*Kocuria* spp., *Staphylococcus* spp., and *Sphingomonas* spp.)^36^. Given that the surface of the canvas and artwork may offer limited supply of nutrients and humidity for bacterial growth and survival, it is not surprising that bacteria with the ability to form resistant phenotypes are the ones obtained from the paintings.

Interestingly, *S. paucimobilis*, a bacterium commonly reported in clinical settings, was isolated from *Musas I*. This bacterium is characterized by a strong adhesion capacity to synthetic surfaces due to its ability to produce exopolymers^37^. Other members of the *Sphingomonas* group have been found in ancient wall paintings^38^, but to our knowledge, this is the first time it has been reported from canvas. Similarly, *K. rizhophila* is increasingly recognized as an emergent human pathogen with high capacity to colonize abiotic surfaces^39^. Moreover, this species has been related with biosorption of heavy metals from soils, suggesting that it may adapt to extreme environments^40^. *Kocuria* and related species have also been previously isolated from art pieces^41^.

In the case of *B. cereus* and *A. acidoterrestris*, they are spore forming Gram positive bacteria with capacity to produce extracellular degradative enzymes. *B. cereus* has been widely known as a foodborne pathogenic and a spoilage microorganism able to synthesize proteases, lipases, lecitinases^42^, quitinases^43^, and pectinases^44^. Similarly, *A. acidoterrestris* is recognized for producing decarboxylases that spoil acidic fruit juices^45^. Nonetheless, this is the first report on *A. acidoterrestris* colonizing an artwork. Both microorganisms could be able to survive in the limiting conditions offered by the paintings’ surface, and they may eventually cause some type of damage to them. In fact, *B. cereus* has been previously isolated from XVII century paintings. It is speculated that it may be responsible for discoloration and other types of alterations^46^. However, additional experiments are necessary to elucidate if these bacteria and other microorganisms are able to have a significant impact on the conservation process of the patrimonial paintings from the NTCR.

#### Environmental conditions

The assessment of current damage and risk of damage of cultural and artistic assets related to climate conditions is often considered from the perspective of works that are directly exposed to the environment, such as historic buildings and statues; nonetheless, this topic is becoming relevant within the context of temperature and humidity changes that is associated with the climate change^47^. The impact of environmental changes is also present on the inside of buildings, threatening artworks and driving attention to consider the influence of climate variability and change in conservation^48^. In tropical areas, where rainfall variability and warm temperatures favor moist environments, the risk of damage of cultural and artistic heritage is even higher. Moist environments in buildings with limited isolation enable the propagation of spores and provide suitable conditions for microbial development. The NTCR is in San José province, an area that exhibits an annual rainfall exceeding 1800 mm/year on average and a mean temperature ranging between 14 and 24 $^{\circ }$C approximately (see Fig. 4C–E). An abundant rainfall during the year and a marked interannual variability contribute to moist conditions in the vicinity of the NTCR. Changes in the space not only are limited to climate variations but also to a specific anthropogenic pressure associated with increasing combustion of fossil fuels. The growth in the number of vehicles that circulate the roads surrounding the NTCR contributes to a rise in the pollution inside the building. Moreover, these meteorological conditions favor the deposition of atmospheric pollutants. Hence, to deliver adequate solutions for the conservation of the artworks that are part of the NTCR, it is of great importance to monitor the external and internal environmental conditions in order to have a better understanding of how these factors are compromise them.

The study reveals a steady and constant shape of the air temperature throughout the time that our stations monitored the environmental conditions. The monitor was specially designed for the examination of this diptych. The diurnal cycles of this variable reach their maximum around 3 p.m. (15:00 h using the 24-h clock method as a reference) and a minimum around 3:00 a.m. (3:00 h using the 24-h clock method as a reference). For this in situ study, measurements were collected for approximately 2 months. This study aims to appreciate if the demeanor of the variable is typical or atypical. The NTCR was compared with other museums such as the Louvre Museum, in Paris; the Metropolitan Museum of Art, New York; and National Palace Museum, Taipei, Republic of China. This comparison showed that the highest mean air temperature corresponds to the NTCR (23,5 $^{\circ }$C) followed by the National Palace Museum (21 $^{\circ }$C)^49^, Louvre Museum (12 $^{\circ }$C)^50^, and finally, the Metropolitan Museum of Art (12 $^{\circ }$C)^51^. In the case of the relative humidity variable, we found almost the same sequence of results: National Palace Museum (82%)^49^, followed by NTCR (72.7%), Metropolitan Museum of Art (67%)^51^, and Louvre Museum (65%)^50^. The most surprising result that emerged from the analyzed data was regarding the carbon dioxide (CO$_{2}$) concentration variable. The NTCR has the highest value for this variable: 570 ppm. The order for the other places is as follows: Metropolitan Museum of Art (465 ppm), National Palace Museum (423 ppm), and the Louvre Museum (414 ppm). The high value for CO$_{2}$ can be attributed to the vehicles that transit the roads surrounding the NTCR as well as to people that visit the room where the paintings reside. These findings need to be cautiously interpreted, as the sample size was a clear limitation. Finally, there is abundant room for future research in this area.

#### Microscopic study of the structure of the artworks and return to origins

Aside from the history and environmental and biological conditions, the comprehension of the paintings’ materials and structure is therefore a crucial aspect that completes our insight of these artworks. Our previous studies provided us with information about the layers and their composition^2^; nonetheless, we carried out a further investigation with the aim of finding more secrets of these paintings.

**Figure 5:**
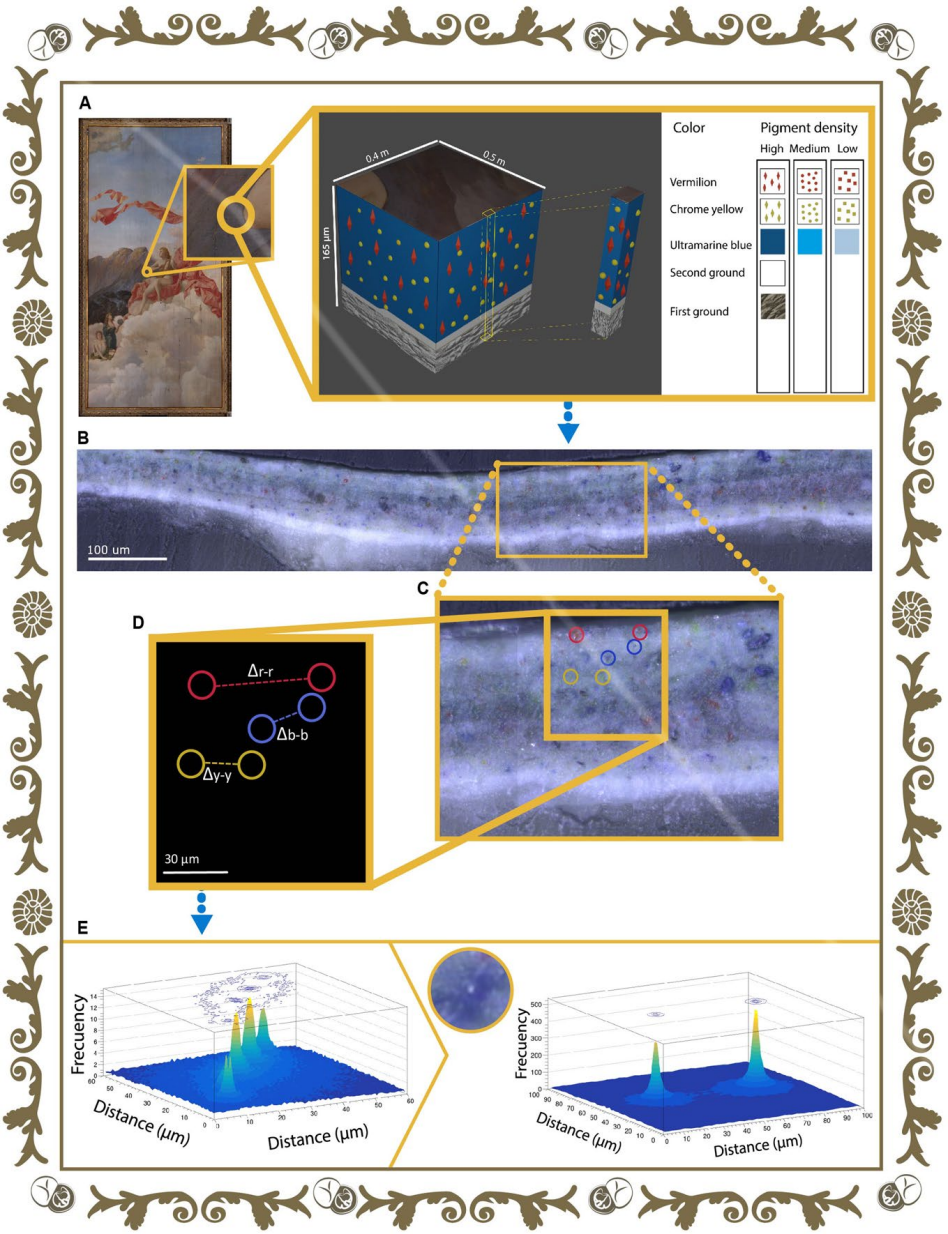
Scheme of the study of the distance between pigments crystals (**A**) First, a sample is extracted from *Musas I* aiming to observe it with the light microscope in order to understand the stratigraphy of the painting, as represented in a 3-coordinate system. (**B**) Then, the microscope images of a region of interest of the cross-section of the sample are used as input into the *PigmentArrangement*^29^ software. (**C**) *PigmentArrangement*^29^ recognizes crystals by colour and carries out (**D**) measurements of the distance between crystals of the same colour ($\Delta$b-b for blue crystals, $\Delta$r-r for red crystals and $\Delta$y-y crystals), and finally, providing (**E**) histograms of the results showing the arrangement of blue pigments in the stratigraphy of the artwork. The figure was created by the authors following what is described in the Supplementary Information and using the following computer tools: Adobe Illustrator, version number 27.1 (https://www.adobe.com/la/products/illustrator.html) and Adobe Photoshop, version number 23.3.2 (https://www.adobe.com/la/products/photoshop.html).

Regarding the painting layers of *Musas I* and *Musas II*, in this work, we sought to establish a proof-of-concept methodology for the understanding of the relationship between the observed colour and the arrangement of the pigments in the colour layers. As can be noted in Fig. 5, the proposed procedure consists of the observation of the cross-section of a specific sample (M1-45B) directly extracted from the painting (Fig. 5A). In the case of M1-45B, it has a dark blue coloration to the naked eye, as it appertains to the paintings’ “mountain area”. For the process, we created a 3D representation of the samples previously studied. This graphic source allows us to outline the information related to the macroscopic colour and the main materials found in that particular area of the painting, see Fig. 5A.

The upper layer corresponds to the paint layer. In this case, the layer is composed of ultramarine, chrome yellow, and vermilion crystals. Below this layer, it is found the second ground that principally contains lead white. The latter pigment has different compositions (hydrocerussite and cerussite), and each one generates distinct effects in the painting. This has been observed in famous works such as Vermeer’s *Girl with a Pearl Earring*^15,52^. Finally, the first ground layer consists of chalk; later, we will discuss this layer (Fig. 5B).

Moving forward, the colour layers are a mixture of binder and pigments that provides colour to the artworks. The software developed, called *PigmentArrangement*^29^, analyzes a selection of photographs that were acquired by microscope (Fig. 5C). *PigmentArrangement* differentiates the different colours of crystals (e.g., blue, red, and yellow) and measures the distance between them (Fig. 5D,E). As a result, histograms of the distances between crystals of the same colour (e.g., blue-blue) and different colours (e.g., blue-red) are obtained (Fig. 5E).

The average distance between the blue crystals (ultramarine), red crystals (vermilion), and yellow ones (chrome yellow) can be observed in the histograms in Fig. 5E. It was expected that blue crystals were going to have the shortest distances between themselves. The reason behind this is that the blue crystals are the ones with a higher amount (approximately 20 crystals in an area of 2000  $\upmu$m$^{2}$). The result was an average distance of 29 $\upmu$m (standard deviation of 12 $\upmu$m) between blue crystals. This matches the hypothesis because the distance for blue crystals is shorter than the distance between the yellow and red crystals, as they have 31 µm (standard deviation of 16 $\upmu$m) and 41 $\upmu$m (standard deviation of 13 $\upmu$m), respectively. As expected, the blue crystals are present in a larger quantity and with shorter distance between themselves. This predominance coincides with the apparent color observed macroscopically in the artwork, from which the sample was taken. This proposed proof-of-concept methodology could be used to explore the dynamics of crystals within layers considering the dimensions (total area of roughly 36.5 m$^{2}$) and the oldness (circa 125 years) of these artworks.

An important limitation of *PigmentArrangement* is its reduced capacity to recognize pigment crystals. If the paint layer has dark colour, difficulties occur when identifying crystals of lighter colours such as yellow. An interrogative arose while analyzing the distances between crystals: have the crystals had this distribution since the painting’s creation? Considering deterioration processes such as the generation of metal soaps in lead pigments, it is unlikely to have the migration and accumulation of material. These two processes are known for changing the microscopic structure^53,54^ and the optical effect of the painting^3,15^. Our new tool *PigmentArrangement* might lead studies specialized in monitoring paintings over time. The changes in crystal distribution could be recorded through a systematic treatment. This software is easy to implement for specific solutions. Ultimately, our research is characterized by taking a novel look at the first ground layer, as it applies a new approach for analyzing the origins of the materials used in *Musas I* and *Musas II*.

#### Materials reveals nannofossils and properties in the painting

Calcareous nannofossils are important algal primary producers in marine environments. Their tests (skeletons) have accumulated over the ocean floors since the Jurassic to recent times^55^. Chalks are calcareous rocks principally composed of the accumulation of these algae. Moreover, chalks are well preserved and exposed in northwestern European seas. This soft white calcareous rock was commonly used as a primer in historical paintings, as it gave a matte and delicate finish.

**Figure 6:**
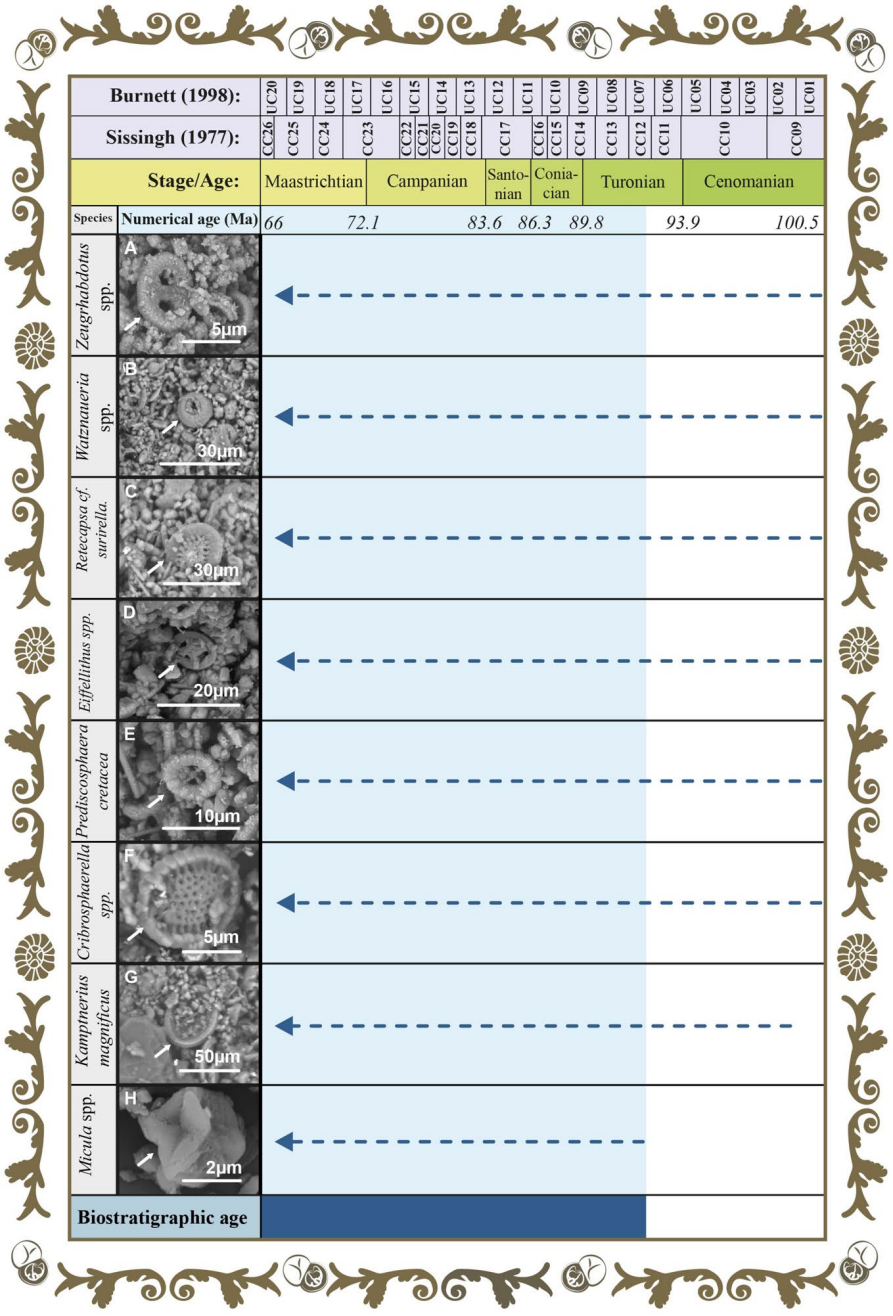
The first ground through the perspective of time: biozonation of the nannofossils of chalk Biostratigraphic ranges of calcareous nannofossils identified in the analyzed scanning electron microcopy images from the first ground of *Musas I* and *Musas II* artworks. The species of calcareous nannofossils correspond to: **(A)** *Zeugrhabdotus* spp., **(B)** *Watznaueria* spp., (**C)** *Retecapsa* cf. *surirella*, **(D)** *Eiffellithus* spp., **(E)** *Prediscosphaera cretacea*, **(F)** *Cribrosphaerella* spp., **(G)** *Kamptnerius magnificus*, and **(H)** *Micula* spp. Light blue bar indicates the biostratigraphic age range. The figure was created by the authors following what is described in the Supplementary Information and using the following computer tools: Adobe Illustrator, version number 27.1 (https://www.adobe.com/la/products/illustrator.html) and Adobe Photoshop, version number 23.3.2 (https://www.adobe.com/la/products/photoshop.html).

The calcareous primer in *Musas I* and *Musas II* was studied, through the analysis of the samples M1-71W, M1-40W, and M2-73W (see sample location in Fig. 3) with the Scanning Electron Microscope (SEM), in order to examine the composition of the first layer. The images showed abundant and well-preserved nannofossils of calcareous nannofossils. The nannofossils were classified using the taxonomic guidelines of previous studies^56,57^ and the biostratigraphic ranges of identified taxa, following the standard low-latitude zonation of Sissingh^58^ and Burnett et al.^59^. The micropaleontological analysis suggests an age of Late Cretaceous. The calcareous nannofossils assemblage observed in the analyzed images consists of *Cribrosphaerella* spp., *Eiffellithus* spp., *Kamptnerius magnificus*, *Micula* spp., *Prediscosphaera cretacea*, *Retecapsa* cf. *surirella*, *Watznaueria* spp., and *Zeugrhabdotus* spp. (Fig. 6). The occurrence of the genus *Micula*, characterized by its first appearance in Biozones UC10 and CC14 and its extinction in Biozones UC20 and CC26, enables us to restrict the age of the material to a biostratigraphic interval ranging from Coniacian to Maastricthian (see Fig. 6)^58,59^. The identified taxa have a wide paleogeographic distribution. Moreover, they have been reported globally at low, middle, and high latitudes^10,60^. This precludes a more detailed analysis of the paleoecological affinities of the nannofossils, at it could work as an indicator of the origin of the materials used in the paintings.

The samples studied came from areas of the painting where the artist used mainly white pigments (Fig. 3A). Calcium carbonate and zinc mainly compose the first ground layer. In the case of the second ground layer, it is primarily constituted of lead white^2^. Although the mix of ground material and white pigment makes possible the white tone, it also provides the painting with some optical properties. The bright effect is a result of the chalk being used as a ground material. It is not only related to the amount of coccoliths that constitutes this material but also to the combination of filters and binders^9,15^. In the diptych, the predominant colour is white^2^ possibly because of the artist’s combination of ground with white pigments (lead white and zinc white) and binders. Therefore, we recommend further studies to ascertain the optical effect present in these paintings. The eight different species of nannofossils discovered in this study could lead to source of the chalk, as it is of great importance for understanding the properties of the material and artist’s intended or desired effect on the painting.

## Conclusions

In conclusion, an interdisciplinary approach based on composition, novel computational tools (*MicroorganismPattern* and *PigmentArrangement*), contamination and/or colonization of microorganisms, and state of conservation have been presented in this research, with the aim of exploring the diptych and obtaining new information about the artist’s artistic process. The proof-of-concept methodology proposed for the monitoring of the environmental conditions and fungal aerial spores in the artworks in company with the software *MicroorganismPattern* establishes a non-invasive approach that could be applied to artworks of worldwide renown. Ultimately, from the experimental data, we try to reveal the detailed nature of the complex artist’s colour palette; our evidence provides emerging insight into Carlo Ferrario’s intentions and creative process, as the ground might be the possible combination of itself with eight nannofossils taxa, white pigments, and binder.

## Methods

The current research accomplished a comprehensive study of two oil paintings—*Musas I* and *Musas II* (ca. 1897) (oil on canvas, 2.96 m by 6.17 m)—of the National Theater of Costa Rica (NTCR). Earlier multi-analytical studies of these artworks allowed a general description of the conservation state, materials, and condition particularities of them^2,61^. Experiments that provide a clear comprehension of the ambient surrounding the artwork, the characterization of its internal parts, the microorganisms living on the air along the painting, and the use of a new software to detect the latter were included in this current exploration.

Furthermore, to describe the pigment’s origin and the layer denominated ground, procedures involving the analysis of the painting’s stratigraphy along with the development of a new software that measures the distance between crystal pigments in the colour layers are consequently required. In the following sections, a description of each experiment performed throughout the investigation is offered.

### Multispectral imaging: photography acquisition

We used visible (VIS) and ultraviolet (UVF) photographs to analyze the biodeterioration areas of interest. The acquisition of the images was taken for previous research published^2,62^ and according to the methodology described by Consentino^63^. We obtain a set of 30 pictures VIS and UVF with a resolution for *Musas I* of 26,004 $\times$ 13,718 pixels and *Musas II* of 25,692 $\times$ 13,711 pixels. We used Cultural Heritage Open Source Pigments Checker (CHSOS) pigment reference for the photos in every region and generated panoramic images in each spectral region using PTGUI Software.

### Software development for biodeterioration recognition

One of the most significant findings that emerged from this study is related to zones of interest for microbiological sampling, as they can be efficiently detected by means of a novel computational tool: *MicroorganismPattern* program^29^. The microorganism’s pattern detection was accomplished using a template matching method. This procedure is an object reveal method that aims to find the position of concrete pixels in a larger image, as these pixels can be equal or similar to the pixels values of the template image. The correlation coefficient template matching method is suited for situations with uneven lighting exposure, as it remains unaffected by localized intensity changes in the images due to the illumination of the place. Additionally, the correlation coefficient tolerates small deviations from the reference pattern^30^.

For the correlation coefficient algorithm, two matrix are established: matrix *R* (template image) and matrix *I* (larger image). *R* and *I* with dimensions *(m x n)* and *(M x N)*, respectively. A third matrix *C* is generated with dimensions *(M-m+1, N-n+1)*. *C* is therefore the correlation coefficient matrix, as it provides the relation between the template *R* and the image *I*. Moreover, the pixels of *C* are denoted as *C(r,s)* (please refer to Fig. 3 to see what these arrays are in our application). The values *C(r,s)* are given by the following equation^30,64^:

1 $$\begin{aligned} C(r,s) = \frac{\sum _{i,j} ([I(i+r,j+s) - \bar{I}][R(i,j)-\bar{R}])}{Z(r,s)}, \end{aligned}$$

with $\bar{R}$ and $\bar{I}$ as the averages of the template image *R* and the subimage of the larger image *I*, respectively. The denominator of the previous equation, *Z(r,s)*, is given by:

2 $$\begin{aligned} Z(r,s) = \sqrt{[\sum _{i,j}(I(i+r,j+s) - \bar{I})^{2}][\sum _{i,j} (R(i,j)-\bar{R})^{2}]}. \end{aligned}$$

The python library OpenCV is used to compute the correlation matrix *C(r,s)* employing the function: TM$\_$CCOEFF$\_$NORMED. The estimates of the correlation matrix *C(r,s)*, Eq. (1), are between the values $-$ 1 and + 1. The pixel values closer to + 1 represent the points where the template image *R* and the larger image *I* are similar. The python library *Scikit-image* is used to find the location of pixels in the *C* matrix whose values are above a selected threshold. The threshold was set in a value where the number of detected areas increases drastically. This occurs for the threshold values in the range (0.65–0.70) for all of the templates used. For this thresholds, the number of detected areas is around ten. If the threshold is reduced, the number of detected areas increases in a factor of six approximately. Subsequently, the OpenCV library is used for drawing boxes, of the size of the corresponding template, on the location of the pixel values above the threshold in the larger image *I*. Before the calculation of the correlation matrix *C(r,s)* using Eq. (1), the rectangular template images of the microorganisms and the images of *Musas I* and *Musas II* are turned into grayscale with OpenCV. The efficiency of the software is determined by taking into consideration the total number of possible areas detected by the program and the number of areas where the sampling returned positive results. For the templates used (*Bacillus cereus*, resolution: 123 $\times$ 156 pixels and *Penicillum*, resolution: 83 $\times$ 95 pixels) the total number of possible detected areas is 18. Out of these areas, only 2 of the sampled areas returned positives results for a efficiency of 11%. It is important to remark that in this first approach, it was not possible to sample all the detected areas by the software. The correlation coefficient method utilized searches in the larger image *I*, a section of the same size and same orientation as the template image *R*^30^. All this information is available to the interested reader at the request of the corresponding author.

### Environmental and microbiological study

#### Isolation of microorganisms

Two sampling dates for isolation of microorganisms were conducted: July 9th, 2021 and July 20th, 2022. To preserve the pictorial artwork, the samples were collected using dry sterile swabs that were circumspectly rubbed over the sections of interest. Sampling sites were previously selected from the patterns formed in the UVF photography and the computer programs developed for this purpose (see below). The entire images of each *Musas I* and *Musas II * paintings were divided into grids according to Fig. 3A. Each grid was subdivided into subsections according to columns called A, B, C y D and rows called 1, 2, 3 to locate the specific site where the sample was taken.

After the collection of samples, the swabs were immersed in 5 mL of 0.1% Sterile Peptone Water and, then, transported at 4 $^\circ$C to the laboratory for further analysis. Each sampling swab was streaked on a specific culture media. For the case of bacteria, Blood Agar (BA) was used; on the other hand, for fungi, Sabouraud glucose agar (SGA) with antibiotics (gentamicin and chloramphenicol) was employed. The BA plates were incubated for 48 hours at 35 $^\circ$C^65^. After incubation, bacterial colonies were microscopically analyzed with Gram staining, and later, they were identified using the VITEK$^{\circledR }$ 2 System (bioMérieux, Marcy-L’Etoile, France). Moreover, colony morphology was assessed as part of the identification process. Each culture was deposited at a temperature of − 80 $^\circ$C in a Brain Heart Infusion Broth supplemented with glycerol.

On the other hand, at 25 $^\circ$C, the SGA plates were nourished and daily assessed for approximately 4 weeks. Subcultures of the different colonial morphotypes were performed on SGA and Potato Dextrose Agar. The purity and preliminary identification of the isolates were determined with lactophenol blue and clear lactophenol for hyaline and black fungi, respectively . Yeasts were identified using the VITEK$^{\circledR }$ 2 System while filamentous fungi employing the MBT (Maldi Biotyper, Bruker Daltonics). Finally, the spectra were analyzed using the Bruker Library and the MSI platform (msi.happy-dev.fr).

#### Spores and air quality

On April 9$^{th}$, 2021, the degree of fungal contamination of ten rooms of the NTCR was determined by measuring the concentration of aerial fungal spores. The former names of the sampled rooms were: Ladies’ Cafeteria, Hall next to the Ladies’ Cafeteria, Men’s Canteen, Hall next to the Men’s Canteen, NTCR Management Office, Foyer (north side), Smoking Room for Ladies, Foyer (south side), Smoking Room for Men, and the Presidential Theater Box. The first five rooms were located on the first floor while the other ones were on the second floor (cf. Supplementary Information).

Burkard Manufacturing Co. Ltd.’s “*Burkard Personal Volumetric Air Sampler*” equipment was used to collect airborne spore particles on glass slides at a rate of 10 L/min^66^. To preserve the integrity of the spores collected by the equipment, they were deposited on a thin and homogeneous layer of petroleum jelly that covered the glass slide. The ideal sampling period for these rooms was 15 min per location. The spores obtained, in situ, were counted and analyzed using a light microscope at 400X in order to examine their structure and to identify them^66,67^.

#### Monitoring of environmental conditions

Novel meteorological stations were built to keep temperature, humidity, light intensity, and CO$_{2}$ content under observation the atmosphere surrounding the artwork pieces. Generally speaking, these low cost and versatile stations internally consist of the following components: (1) SCD30 sensor (manufacturer Sensirion AG) in order to measure the concentration of atmospheric CO$_{2}$, the temperature and relative humidity of the environment, as well as an (2) TSL2591 sensor ((manufacturer Adafruit) to accomplish light intensity measurements. Next to these sensors is a (3) DS3231 RTC (Real Time Clock) module to print a time stamp on them. These components communicate and send data to (4) the Feather M0 Adalogger microcontroller (manufacturerAdafruit), via the I2C bus, and then stored in a SD card. Regarding the structure of the stations, it was designed and tested using the commercial tool SolidWorks$^{\textrm{TM}}$ and finally printed in 3D, seeking to optimize the size and internal distribution of the components. The employment of these stations on the surfaces of the NTCR and its internal structure were carried out to answer qualitative and quantitative methodological questions of the previously established variables. Nonetheless, several iterations were designed and constructed before the final design was approved by the restorers of the NTCR. The final 3D-prototype has a structure that prioritizes a functional distribution between the microcontroller, the battery, and the sensors. The prototype was also built considering a harmonious design for the NTCR environment.

Turning now to the location of the meteorological stations, it is important to mention that they were on top of the two door frames of the room where the NTCR displays the artworks. Throughout the whole day, our novel stations monitored the atmospheric conditions. The data is recorded on SD chips and it is approximately retrieved every month. In order to guarantee data fidelity and measurement continuity during the retrieval process, the stations’ batteries are recharged and the data is analyzed. The data is collected from the SD chips in a CSV file format. These files are analyzed using a Python script as it graphs how a specific variable varies in relationship to time over the sampling period.

### Pigments study

#### Optical microscope observation of pigments

With the aim of studying the distribution of the pigments in the crystals, a sample of the painting *Musas I* was analyzed (M1-45B, see sample location in Fig. 3). The sampling and cross-section preparation procedures of this sample are described in our previous research of this painting^2^.

In the present investigation, micrographs of the cross-section of the sample were obtained using a microscope Witec Alpha 300 with a CCD camera and objective lens of 100x. To cover an area of approximately 160,000 $\upmu$m$^{2}$, around of 75 micrographs were acquired for the cross-section. To obtain these photos, the micrometers of the microscope’s stage were moved maintaining a known distance, as it allows the adjacent photos to overlap in the vertical and horizontal direction. Subsequently, the photos were arranged in rows, and after that, each photo in the row was arranged in columns. Each column in a specific row could have from two to four pictures, approximately. Those pictures were merged using Adobe Photoshop$^{\circledR }$ to have just one photo for a column in a specific row.

To computationally recreate the layers of the painting, microscope images were observed using the commercially available Blender, a 3D modeling and free access software. A cube representation that has information of the amount and average thickness of the layers as well as the density of pigment present in the sample was created. The thickness of the layers was amplified in order to be able to observe them in greater detail. To clarify, the sampling is only performed in specific sectors of the painting; for this reason, the missing data of the remaining sectors is interpolated.

#### Software development for pigments arrangement

The code used for analyzing *PigmentArrangement*^29^ was developed in Python3 programming language, applying libraries such as NumPy, Matplotlib, OpenCV, and Scipy in a Linux Ubuntu 16.04.4 virtual machine.

The software aims to perform an analysis of pigment arrangement in the samples obtained from *Musas I* and *Musas II*. Studying the average distance between the crystals of different pigments, histograms and data tables were obtained with the values for the average distance between crystals of the same colour (e.g., blue–blue, red–red) and combination of colours (e.g., blue–red, blue–yellow). For the analysis of the average distance, the closest images in the horizontal and vertical direction are considered, but more specifically, the crystals located in the images on the left, right, above, and below the one being analyzed.

Essentially, the software consists of codes that allow cuts in the photographs as well as perform a colour scanning depending on the pigments found in the samples. An analysis must be performed to find the average common area between the images of the samples. The analysis result is an index used to decrease the percentage of error in the calculation of every distance and in the average of them. All this information is available to the interested reader at the request of the corresponding author.

### Material origins

The first ground layers of *Musas I* and *Musas II*^2^ were analyzed with the aim of describing the material and its possible provenance. Three samples identified as M2-73W, M1-71W, and M1-40W were studied (see sample location in Fig. 3). The fragments (1–2 mm) were directly observed using a Hitachi SEM S-3700. Backscattered electrons imaging and elemental analyses were primarily obtained. Moreover, the underground layer contains calcareous nannofossils. To characterize and analyze the biogenic content, a small fragment of the sample was placed in an Eppendorf tube with 0.5 mL of ultrapure water, and then, it was sonicated for 10 min. Afterwards, in a labeled glass slide, one drop of the watery residue was deposited. Then, it was dried for a couple of minutes on a white-hot plate. Later, the samples were sputter coated with a 50 nm layer of gold in an EMS-150RS-4 Ion Coater, and images were taken with a SEM Zeiss Sigma 300. At last, the nannofossils were classified using the taxonomic guidelines^68^ and biostratigraphic ranges of identified taxa following the standard low-latitude zonations^58,59^.

To study the possible origins of the pigments identified in *Musas I* and *Musas II*^2^, bibliographical and geographical research was done ^17–28^. Each pigment was identified and categorized according to their age, use, trade route, and chemical composition. After matching the chemical compositions between the pigments and the minerals as well as their correlation with specific geographical information appertaining to the artist’s locality in addition to the mineral extraction sites during the 19th century, it was possible to triangulate mineral extraction sites where the minerals of interest are present.

## Supplementary Information

Supplementary Information.

## Data Availability

All data needed to evaluate the conclusions in the manuscript are present in the manuscript and/or the Supplementary Information.
