# Publisher Correction: Multidisciplinary approach to the study of large-format oil paintings

**DOI:** 10.1038/s41598-023-31171-0

**Published:** 2023-03-10

**Authors:** P. Calderón-Mesén, D. Jaikel-Víquez, M. D. Barrantes-Madrigal, J. Sánchez-Solís, J. P. Mena-Vega, J. Arguedas-Molina, K. Ureña-Alvarado, G. Maynard-Hernández, L. Santamaría-Montero, M. Cob-Delgado, E. Angulo-Pardo, Felipe Vallejo, M. I. Sandoval, A. M. Durán-Quesada, M. Redondo-Solano, O. A. Herrera-Sancho

**Affiliations:** 1 Centro de Investigación en Estructuras Microscópicas, Universidad de Costa Rica, 2060 San Pedro, San José Costa Rica; 2 Instituto de Investigaciones en Arte, Universidad de Costa Rica, 2060 San Pedro, San José Costa Rica; 3 Facultad de Microbiología, Universidad de Costa Rica, 2060 San Pedro, San José Costa Rica; 4 Centro de Investigación en Enfermedades Tropicales (CIET), Universidad de Costa Rica, 2060 San Pedro, San José Costa Rica; 5 Escuela de Química, Universidad de Costa Rica, 2060 San Pedro, San José Costa Rica; 6 Escuela de Ingeniería Eléctrica, Universidad de Costa Rica, 2060 San Pedro, San José Costa Rica; 7 Escuela de Física, Universidad de Costa Rica, 2060 San Pedro, San José Costa Rica; 8 Diseño Gráfico, Sede de Occidente, Universidad de Costa Rica, 2060 San Pedro, San José Costa Rica; 9 Department of History of Art, Cornell University, Ithaca, NY 14853 USA; 10 Escuela de Artes Plásticas, Universidad de Costa Rica, 2060 San Pedro, San José Costa Rica; 11 Instituto Costarricense de Investigación y Enseñanza, en Nutrición y Salud, 42250 Cartago, Costa Rica; 12 Grupo de Investigaciones en Estratigrafía, y Vulcanología (GIEV-Cumanday) y Departamento de Ciencias Geológicas de la Universidad de Caldas, Instituto de Investigaciones en Estratigrafía (IIES), Calle 65 # 26-10, 1700004 Manizales, Colombia; 13 Departamento de Geología, Facultad de Ciencias, Universidad de Salamanca, Spain, Plaza de los Caídos, S/N, 37008 Salamanca, Spain; 14 Escuela Centroamericana de Geología, Universidad de Costa Rica, 2060 San Pedro, San José Costa Rica; 15 Departamento de Física Atmosférica, Oceánica y Planetaria & Laboratorio para la Observación del Sistema Climático, Escuela de Física, Universidad de Costa Rica, 2060 San Pedro, San José Costa Rica; 16 Centro de Investigación en Contaminación Ambiental, Universidad de Costa Rica, 2060 San Pedro, San José Costa Rica; 17 Laboratorio de Investigación y Entrenamiento en Microbiología de Alimentos y Aguas (LIMA), Universidad de Costa Rica, 2060 San Pedro, San José Costa Rica; 18 Centro de Investigación en Ciencias Atómicas Nucleares y Moleculares, Universidad de Costa Rica, 2060 San Pedro, San José Costa Rica

Correction to: *Scientific Reports* 10.1038/s41598-023-28777-9, published online 07 February 2023

The original version of this Article contained errors in the Affiliations. P. Calderón-Mesén, M. D. Barrantes-Madrigal, J. Arguedas-Molina, and O. A. Herrera-Sancho were incorrectly affiliated with ‘Facultad de Microbiología, Universidad de Costa Rica, 2060, San Pedro, San José, Costa Rica’. In addition, D. Jaikel-Víquez and M. Redondo-Solano were incorrectly affiliated with ‘Escuela de Química, Universidad de Costa Rica, 2060, San Pedro, San José, Costa Rica’.

The correct affiliations are listed below.

**P. Calderón-Mesén**

Centro de Investigación en Estructuras Microscópicas, Universidad de Costa Rica, 2060, San Pedro, San José, Costa Rica

Instituto de Investigaciones en Arte, Universidad de Costa Rica, 2060, San Pedro, San José, Costa Rica

**M. D. Barrantes-Madrigal**

Instituto de Investigaciones en Arte, Universidad de Costa Rica, 2060, San Pedro, San José, Costa Rica

Escuela de Química, Universidad de Costa Rica, 2060, San Pedro, San José, Costa Rica

**J. Arguedas-Molina**

Instituto de Investigaciones en Arte, Universidad de Costa Rica, 2060, San Pedro, San José, Costa Rica

Escuela de Química, Universidad de Costa Rica, 2060, San Pedro, San José, Costa Rica

**O. A. Herrera-Sancho**

Instituto de Investigaciones en Arte, Universidad de Costa Rica, 2060, San Pedro, San José, Costa Rica

Escuela de Física, Universidad de Costa Rica, 2060, San Pedro, San José, Costa Rica

Centro de Investigación en Ciencias Atómicas Nucleares y Moleculares, Universidad de Costa Rica, 2060, San Pedro, San José, Costa Rica

**D. Jaikel-Víquez**

Instituto de Investigaciones en Arte, Universidad de Costa Rica, 2060, San Pedro, San José, Costa Rica

Facultad de Microbiología, Universidad de Costa Rica, 2060, San Pedro, San José, Costa Rica

Centro de Investigación en Enfermedades Tropicales (CIET), Universidad de Costa Rica, 2060, San Pedro, San José, Costa Rica

**M. Redondo-Solano**

Instituto de Investigaciones en Arte, Universidad de Costa Rica, 2060, San Pedro, San José, Costa Rica

Facultad de Microbiología, Universidad de Costa Rica, 2060, San Pedro, San José, Costa Rica

Centro de Investigación en Enfermedades Tropicales (CIET), Universidad de Costa Rica, 2060, San Pedro, San José, Costa Rica

Laboratorio de Investigación y Entrenamiento en Microbiología de Alimentos y Aguas (LIMA), Universidad de Costa Rica, 2060, San Pedro, San José, Costa Rica

As a result, the Affiliations have been renumbered.

The original Article and accompanying Supplementary Information file have been corrected.

