# Supplementary material for: Multidisciplinary approach to the study of large-format oil paintings
Source: Sci Rep. 2023 Feb 7;13:2143. doi: 10.1038/s41598-023-28777-9 (PMC9905513; doi:10.1038/s41598-023-28777-9)
Supplement: Supplementary file 1 — Supplementary Information. [file 41598_2023_28777_MOESM1_ESM.pdf]

Multidisciplinary approach to the study of artworks: environmental conditions, colour palette, microbial colonization and nannofossils in *Las Musas* painting

P. Calderón-Mesén[1,2,+], D. Jaikel-Víquez[2,3,4,+], M.D. Barrantes-Madrigal[2,5,+], J. Sánchez-Solís[6], J. Mena-Vega[7], J. Arguedas-Molina[2,5], K. Ureña-Alvarado[8], G. Maynard-Hernández[7], L. Santamaría-Montero[9,10], M. Cob-Delgado[11], E. Angulo-Pardo[12], Felipe Vallejo[12,13], M.I. Sandoval[14], A. M. Durán-Quesada[15,16], M. Redondo-Solano[2,3,4,17], and O.A. Herrera-Sancho[2,7,18,*]

[1]Centro de Investigación en Estructuras Microscópicas, Universidad de Costa Rica, 2060 San Pedro, San José, Costa Rica
[2]Instituto de Investigaciones en Arte, Universidad de Costa Rica, 2060 San Pedro, San José, Costa Rica
[3]Facultad de Microbiología, Universidad de Costa Rica, 2060 San Pedro, San José, Costa Rica
[4]Centro de Investigación en Enfermedades Tropicales (CIET), Universidad de Costa Rica, 2060 San Pedro, San José, Costa Rica
[5]Escuela de Química, Universidad de Costa Rica, 2060 San Pedro, San José, Costa Rica
[6]Escuela de Ingeniería Eléctrica, Universidad de Costa Rica, 2060 San Pedro, San José, Costa Rica
[7]Escuela de Física, Universidad de Costa Rica, 2060 San Pedro, San José, Costa Rica
[8]Diseño Gráfico, Sede de Occidente, Universidad de Costa Rica, 2060 San Ramón, Alajuela, Costa Rica
[9]Department of History of Art, Cornell University, Ithaca, NY, 14853
[10]Escuela de Artes Plásticas, Universidad de Costa Rica, 2060 San Pedro, San José, Costa Rica
[11]Instituto Costarricense de Investigación y Enseñanza, en Nutrición y Salud, 42250 Cartago, Costa Rica
[12]Instituto de Investigaciones en Estratigrafía (IIES), Grupo de Investigaciones en Estratigrafía, y Vulcanología (GIEV-Cumanday) y Departamento de Ciencias Geológicas de la Universidad de Caldas, Calle 65 # 26-10, 1700004 Manizales, Colombia
[13]Departamento de Geología, Facultad de Ciencias, Universidad de Salamanca, España. Plaza de los Caídos, s/n, 37008 Salamanca
[14]Escuela Centroamericana de Geología, Universidad de Costa Rica, 2060 San Pedro, San José, Costa Rica
[15]Departamento de Física Atmosférica, Oceánica y Planetaria & Laboratorio para la Observación del Sistema Climático, Escuela de Física, Universidad de Costa Rica, 2060 San Pedro, San José, Costa Rica
[16]Centro de Investigación en Contaminación Ambiental, Universidad de Costa Rica, 2060 San Pedro, San José, Costa Rica
[17]Laboratorio de Investigación y Entrenamiento en Microbiología de Alimentos y Aguas (LIMA), Universidad de Costa Rica, Costa Rica
[18]Centro de Investigación en Ciencias Atómicas Nucleares y Moleculares, Universidad de Costa Rica, 2060 San Pedro, San José, Costa Rica
*oscar.herrerasancho@ucr.ac.cr
+these authors contributed equally to this work

This PDF file includes:
Table S1
Figure S1
Concentration of fungal aerial spores
Description of the creative process in the design of the figures

Concentration of fungal aerial spores

In Table S1, we present the concentration and distribution of aerial fungal spores of ten rooms from the TNCR. Over 94% of the spores identified were characterized as *Cladosporium* (62,92%), Ascospores (18,02%), *Aspergillus/Penicillium* (9,58%), and Basidiospores (3,86%). This is consistent with other volumetric aerial analysis performed in Costa Rica's Metropolitan

Area[75,76]. The rooms with the highest concentration of Ascospores and Basidiospores were the Ladies's Cafeteria and the Hall attached to the Ladies's Cafeteria. However, there is no surprise for this result, as these rooms are currently being used as the theater's coffee shop -a place constantly being visited by people-. For this reason, the windows of those rooms are opened throughout the day. This allows fungal spores from surrounding vegetation to enter the premises. Due to the COVID-19 pandemic, the other rooms were closed for over a year before the sampling date. Hence, for those places, we only identified fungal spores that are associated with interiors and are able to grow and accumulate on walls, ceilings, and air conditioners[75,76].

Supplementary Table S1. Atmospheric fungal spores in and around the diptych space Concentration of fungal aerial spores per cubic meter in ten rooms of the National Theater of Costa Rica, April 9th 2021.

**Type of spore**	**Fungal spore concentration (spore per cubic meter)**									
	**Ladies's Cafeteria**	**Hall attached to the Ladies's Cafeteria**	**Men's Canteen**	**Hall attached to the Men's Canteen**	**NTCR Management Office**	**Foyer (North Side)**	**Foyer (South Side)**	**Smoking Room for Ladies**	**Smoking Room for Men**	**Presidential Theater Box**
Alternaria	0	0	0	0	0	0	0	8	0	0
Ascospores	293	460	387	220	433	200	183	175	150	175
Aspergillus/ Penicillium	93	120	21	213	140	73	133	133	125	125
Basidiospores	227	60	40	28	40	27	67	58	8	20
Cercospora	7	7	0	7	14	0	0	8	0	0
Cladosporium	1647	1100	1293	440	3100	660	450	317	592	1733
Curvularia	7	21	7	0	21	0	0	8	17	0
Fusarium	47	21	0	7	27	0	33	17	25	0
Helminthosporium	0	0	0	0	7	0	0	0	0	0
Nigrospora	0	14	0	0	0	0	8	0	0	0
Pithomyces	0	7	14	7	7	0	0	0	0	0
Smuts	14	40	0	21	27	20	25	8	8	58
Stachybotrys	0	7	0	0	21	7	8	8	0	0
Torula	0	0	0	7	0	0	8	0	0	0
*Not identified**	7	7	14	14	21	14	0	17	25	8
**Total**	**2342**	**1864**	**1776**	**964**	**3858**	**1001**	**915**	**757**	**950**	**2119**

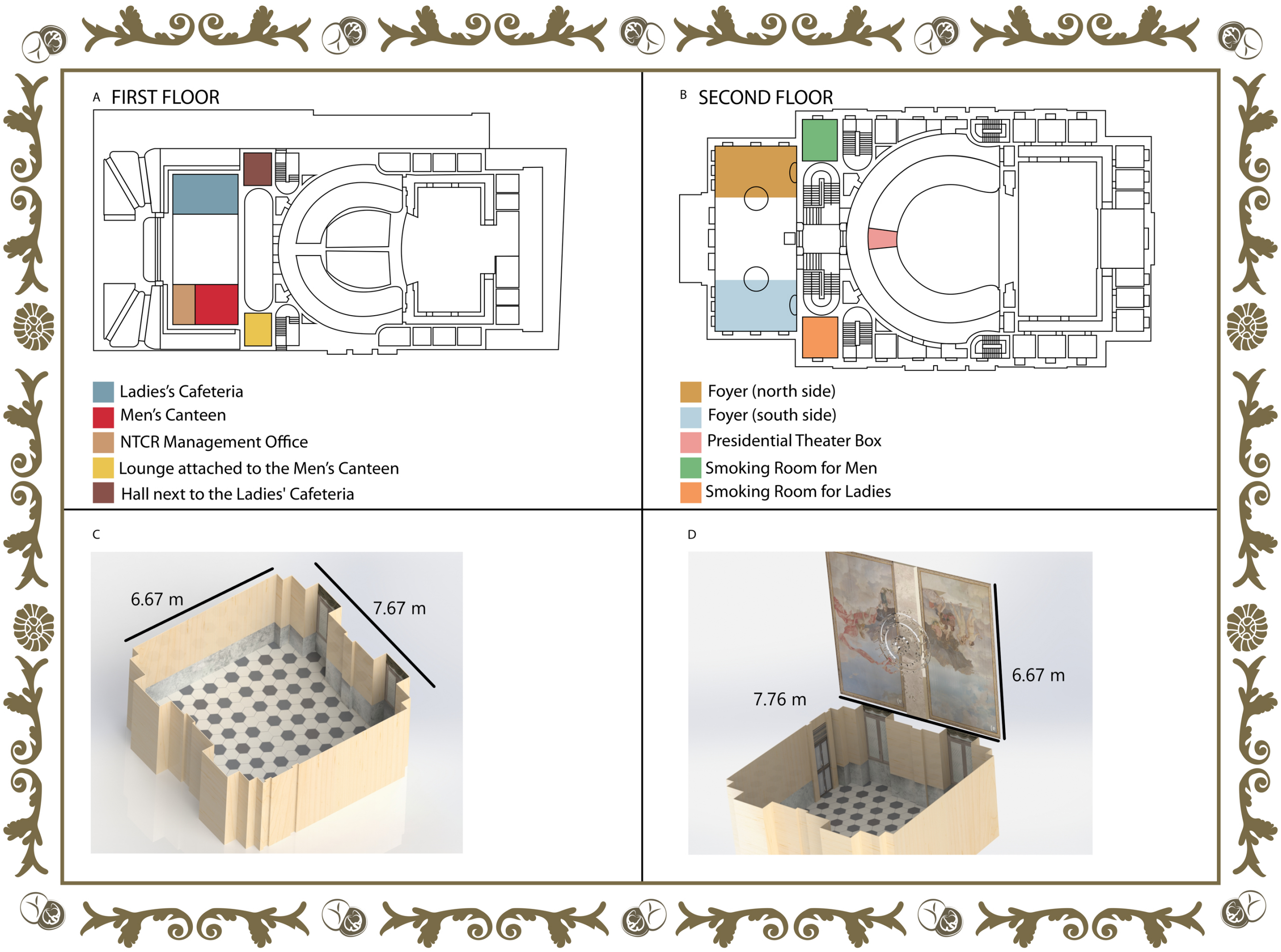

Supplementary Figure S1. Layout distribution of the rooms sampled for fungal aerial contamination in the TNCR A volumetric air sample technique was applied to determine the concentration of aerial fungal spores in ten different rooms of the TNCR. The distribution of the rooms located in the first floor is shown in panel (A) and the ones in the second floor in panel (B). Panels (C and D) present the layout of the rendering and the measurements of the room formerly named Men's Canteen, where *Musas I* and *II* are located in its ceiling. The figure was created by the authors following what is described in the Supplementary Information and using the following computer tools: Adobe Illustrator, version number 27.1 (https://www.adobe.com/la/products/illustrator.html) and Adobe Photoshop, version number 23.3.2 (https://www.adobe.com/la/products/photoshop.html).

Artistic design in the figures and decorative frame representation

The purpose of this section is to define the artistic design used in the figures and describe the decorative frame representation. All the figures were prepared with information from all the authors of the different methodologies used and finally using the following computer tools that belong to the University of Costa Rica: Adobe illustrator, version number 27.1 (https://www.adobe.com/la / products/illustrator.html) and Adobe Photoshop, version number 23.3.2 (https://www.adobe.com/la/products/photoshop.html). In the case of Fig. 2, the aspects already established for Fig. 1 were taken as reference. The latter was designed by the graphic designer Fabiola Salas Barahona. Moreover, the color palette, the shapes' outlining, and the digital manual technique were applied and designed by the person previously mentioned. Fig. 3, 4, 5, 6 and S1 have the same digital intervention in their design. For this reason, these figures have the same color palette used in Fig. 1. Furthermore, to create a visual unity, a frame in golden color was added to all figures. Regarding the composition in Fig. 2, the main structure is a centripetal spiral. Within this spiral, an irregular chronological path is drawn. This figure starts in panel A with visual information of Greek mythology. The information for this panel was taken from Fig. 1, but some modifications were made. Fig. 2 ends in panel H with a zoom of the stylized version of the paintings *Musas I* and *Musas II*. In this panel, a synthesis of the environmental conditions surrounding the artwork is presented, as it is of great importance in order to determine the deterioration process. In the case of Fig. 3, 4, 5, and 6, they were digitally assembled with the information -photographs and graphs- provided by the researchers. These figures illustrate the investigation process and the main results.

On the other hand, the frame's design was created taken into consideration some of the results obtained during the investigation, as it is the case for the nannofossils *Prediscosphaera cretacea* (Fig. 6E) along with the fungi *Cladosporium halotolerans* with *Cladosporium dominicanum* (Fig. 3G). Additionally, decorative elements from the Rococo style were used on it. Finally, a golden color was applied in order to mirror the paintings' frame.
